# Psychiatric Emergencies in Los Angeles County During, and After, Initial COVID-19 Societal Restrictions: An Interrupted Time-Series Analysis

**DOI:** 10.1007/s10597-022-01043-4

**Published:** 2022-12-12

**Authors:** Tim A. Bruckner, Shutong Huo, Michael Huynh, Senxi Du, Andrew Young, Annie Ro

**Affiliations:** 1 Program in Public Health, University of California, Irvine, Irvine, USA; 2 David Geffen School of Medicine, University of California, Los Angeles, Los Angeles, USA; 3 Harbor UCLA Medical Center, University of California, Los Angeles, Los Angeles, USA

**Keywords:** Psychiatric emergency room visits, COVID-19 pandemic, Societal restrictions, Mental health services

## Abstract

Emergency department (ED) visits for psychiatric care in the US reportedly declined during the COVID-19 pandemic. This work, however, does not control for strong temporal patterning in visits before the pandemic and does not examine a potential “rebound” in demand for psychiatric care following the relaxation of initial societal restrictions. Here, we examine COVID-19-related perturbations in psychiatric care during and after the 1st stage of societal restrictions in the largest safety-net hospital in Los Angeles. We retrieved psychiatric ED visit data (98,888 total over 156 weeks, Jan 2018 to Dec 2020) from Los Angeles County + USC Medical Center. We applied interrupted time series methods to identify and control for autocorrelation in psychiatric ED visits before examining their relation with the 1st stage of societal restrictions (i.e., March 13 to May 8, 2020), as well as the subsequent “rebound” period of relaxed restrictions (i.e., after May 8, 2020). Psychiatric ED visits fell by 78.13 per week (i.e., 12%) during the 1st stage of societal restrictions (SD = 23.99, p < 0.01). Reductions in ED visits for alcohol use, substance use, and (to a lesser extent) anxiety disorders accounted for the overall decline. After the 1st stage of societal restrictions, however, we observe no “rebound” above expected values in psychiatric ED visits overall (coef = − 16.89, SD = 20.58, p = 0.41) or by diagnostic subtype. This pattern of results does not support speculation that, at the population level, foregoing ED care during initial societal restrictions subsequently induced a psychiatric “pandemic” of urgent visits.

## Introduction

The arrival of documented COVID-19 cases in the US in winter 2020 stimulated a wide array of societal restrictions designed to reduce the spread of person-to-person transmission. These measures, initiated at the federal, state, and local levels in March and April 2020, aimed to limit population movement and contact with persons outside the home. For instance, Los Angeles County (LAC) ordered on March 16, 2020, the closure of public schools, bars, gyms, entertainment centers, and indoor dining (County of Los Angeles Department of Public Health, 2020).

Given the large and widespread disruption to routine social and economic activity, as well as uncertainty regarding the course of COVID-19 infection rates, scholars predicted a “psychiatric pandemic” in which help-seeking for several conditions (e.g., suicidal ideation, anxiety, mood, and substance use disorders) would subsequently increase above expected levels (Gunnell et al., 2020; Reger et al., 2020; Vigo et al., 2020). Counter to expectations, however, in the US and elsewhere, visits for emergency psychiatric care fell in spring 2020 (Holland et al., 2021). In addition, a report on three specialty psychiatric emergency centers in the US finds an 8 to 9 percent drop in psychiatric emergencies after March 15, 2020 (Simpson et al., 2021). This decline coheres with the broader observation nationwide of avoidance of health care visits unrelated to COVID-19. For instance, surveillance data from selected US hospitals indicate a 42% decline in the emergency department (ED) visits overall in March and April 2020 compared to the same period in 2019 (Hartnett et al., 2020; Holland et al., 2021).

The literature documenting declines in psychiatric ED visits following COVID-19 in the US remains limited in two key ways. First, the intensity and scope of societal restrictions differ substantially over time. Whereas 43 of 50 states imposed some form of “stay-at-home” order or restrictions on social and economic activity, the majority of states lifted restrictions by mid-May 2020, given that SARS-CoV-2 prevalence appeared low (Das et al., 2022). The lifting of state-imposed restrictions could have stimulated a return to pre-pandemic levels of help-seeking in the health care setting, suggesting that the short-term ED utilization decline has little bearing on long-term mental health utilization trends. Alternatively, foregoing care during the first phase of societal restrictions (i.e., March to May 2020) could have exacerbated conditions in ways that subsequently increased help-seeking for psychiatric ED care *above* pre-pandemic levels. This elevated use could arise from two sources: the truly ill deferred needed care during the early days of the pandemic, or the curtailed social and economic activity during the shutdown created more mental health problems that contributed to higher demand once the societal shutdowns lifted. The literature includes no test of this potential rebound. In addition, we do not know whether psychiatric ED visits differed from statistically expected levels during each of the two distinct periods: the initial phase of the restrictions (i.e., March 13 to May 8, 2020) and the “post”-restriction era (i.e., after May 8, 2020).

Second, recent population-level work suggests that the COVID-19 pandemic preceded increases in some mental health symptoms but decreases in others. In a study of 8 million helpline calls from 19 countries, Brülhart and colleagues found that calls related to fear and anxiety increased, while those for suicidal ideation and substance use decreased within six weeks of the initial pandemic (Brülhart et al., 2021). Diagnostic subtypes of psychiatric ED visits may therefore show distinct**—**and potentially countervailing**—**responses to phases of COVID-19 restrictions. For this reason, in aggregate, prior examinations of psychiatric ED visits may obscure important subtype differences.

We address these two limitations directly by examining psychiatric ED visits at the largest health care provider in LAC, the most populous county in the US. Unlike earlier work, we separate potential “shelter-in-place” from “rebound” responses by applying rigorous time-series methods (Bernal et al., 2017) which researchers increasingly use to examine the influence of interruptions such as COVID-19 (Bernal et al., 2017; Bruckner et al., 2019; Catalano et al., 2021). Another improvement relative to earlier work in this area is that we focus on a region that imposed major stay-at-home restrictions over a clearly defined eight-week period. This circumstance minimizes measurement error in assessing help-seeking responses when using larger regions with a heterogeneous mix of lenient and strict COVID-19 policies at the same time.

We hypothesize that all-cause psychiatric ED visits decreased during the initial shutdown relative to the pre-pandemic level due to the public health restrictions on movement and activity. We also focus on the post-restriction period separately to test whether psychiatric ED visits “rebound” above expected levels after the relaxation of restrictions. If we find that ED visits rose above pre-pandemic levels, this could support the notion of a “psychiatric pandemic” induced by societal disruption and uncertainty. Finally, if we observe a statistically detectable change in psychiatric ED visits, we then examine which subtypes of conditions may have been more or less adversely affected during that period.

## Methods

### Data Sources

We analyzed all psychiatric ED visits to the LAC + USC Medical Center (LAC + USC) between January 5th, 2018, and December 31st, 2020. LAC + USC is the largest public hospital facility in Los Angeles County. The facility provides a full spectrum of emergency, inpatient, and outpatient services, including psychiatric services. The ED at LAC + USC ranks as one of the busiest in the US that it averages over 150,000 ED visits per year (“30 Hospitals with the Most ER Visits,” n.d.). As a public hospital, LA + USC largely serves Hispanic/Latino patients, Medicaid-insured patients and uninsured patients, but also provides care to uninsured and privately insured populations. The ED for psychiatric emergencies in LAC + USC has a separate area. If patients present with psychiatric emergencies without a medical emergency, they are often treated there and may be discharged or admitted to a psychiatric facility.

Our data come from the LAC + USC Medical Center’s Vizient Health System Data, a hospital billing and administrative claims database that records all medical center patient visits. Vizient Health System Data provided us with a “clean” dataset that already removed records with missing data. Each individual patient can contribute to multiple ED encounters. We used all encounters from the database. We did not apply any restrictions (e.g., by age or insurance type) when aggregating ED counts.

### Outcome

We identified a psychiatric ED visit using Clinical Classification Software (CCS). Consistent with the literature, we considered the ICD-9 (International Classification of Diseases, Ninth Revision) code as a psychiatric ED visit if *any* diagnosis for a visit listed a corresponding CCS code ranging from 650 to 670 (“HCUP-US Home Page,” n.d.) (Hall et al., 2018). We classified visit type according to six CCS categories (i.e., schizophrenia, mood disorders, anxiety disorders, suicide and self-inflicted injury, alcohol use disorders, substance use disorders.

We used the count of psychiatric ED visits as the dependent variable. The records include both “treat and release” outpatient ED visits as well as ED visits that ultimately result in an inpatient stay. We aggregated psychiatric ED visits into 7-day periods. Patients may appear more than once in any week, given the visit-level nature of the dataset. We summed ED visits starting on Fridays and ending on Thursdays so that Friday, March 13th, 2020, the date that the Trump Administration declared a national emergency due to COVID-19, serves as the first day of the exposed “anchor” 7-day period. On Monday, March 16th, 2020, moreover, LA County ordered the closure of public schools, bars, gyms, entertainment centers, and indoor dining. This circumstance led us to examine 156 full weeks of ED visits beginning January 5, 2018, and ending December 31, 2020. These 7-day periods, which we refer to as weeks for simplicity, represent the longest time series available to us at the time of our test.

The key independent variable is the 1st societal shutdown in LA County, coded as “1” for the eight weeks from March 13 to May 8, 2020, and “0” for all other weeks. We specified this 1st shutdown as the exposure because it coincided with a substantial and unexpected perturbation to routine activities. All project activities were reviewed and approved by the USC Institutional Review Board (HS-19–00890), which served as a reliance for the UC Irvine Institutional Review Board. All data were de-identified to conform to Health Insurance Portability and Accountability Act (HIPAA) requirements.

### Analysis

We follow the tradition of the interrupted time series analysis and employ autoregressive, integrated, moving average routines (ARIMA) recommended in the literature (Bernal et al., 2017; Box et al., 2016; Catalano & Serxner, 1987). Psychiatric ED visits may exhibit well-characterized temporal patterns, including seasonality, trend, and the tendency for high or low values to be “remembered” into subsequent months. These patterns complicate correlational tests because the expected value of a patterned series is not its mean.

To address this autocorrelation issue, we used methods devised by Box and Jenkins (Box et al., 2016; Catalano & Serxner, 1987). These routines express autocorrelation as a product of “autoregressive” (AR), “integrated” (I), and “moving average” (MA) parameters, collectively referred to as ARIMA models. After identification and control for autocorrelation, the analyst then inserts the “interruption” exposure variable to determine whether the interruption correlates with the residual (i.e., unpatterned) values of the dependent variable.

We implemented the above time-series approach with the following steps. First, for the 114 weeks of psychiatric ED visits leading up to, but not including, COVID-19 restrictions (i.e., Jan 5, 2018, to March 12, 2020), we identified potential AR, I, or MA parameters. Second, we added ARIMA parameters to express autocorrelation identified in its residual values (i.e., error term). Third, we estimated the ITS model formed by adding the first stay-at-home orders restriction binary variable into the model resulting from step 2. We specified a synchronous relation (i.e., psychiatric ED visits fall during the first stay-at-home orders) and, unlike step 1, used *all* 156 weeks of the entire series (i.e., to Dec 31, 2020). Fourth, we inspected the residuals of the time-series equation to ensure that they exhibited no autocorrelation. Fifth, we assessed the stability of results to outliers (Chang et al., 1988). We used software from Scientific Computing Associates (version 5.4.6, SCA Corp., Villa Park, IL).

After May 8th, 2020, the State of California relaxed some restrictions (including re-opening retail businesses). To explore this potential rebound effect after the first shutdown, we performed another ITS test by classifying all weeks from May 9th 2020 to the last week of the study period (i.e., to Dec 31st, 2020) as “1” and all other weeks as “0.” In this specification, we did not control for the first stay-at-home order when estimating the “rebound” coefficient. Lastly, we explored whether any weeks from May 9th 2020, and thereafter indicated potential positive outliers in psychiatric ED visits, which may indicate punctuated epochs of significant “rebounds” in need for psychiatric ED care.

## Results

**Fig. 1:**
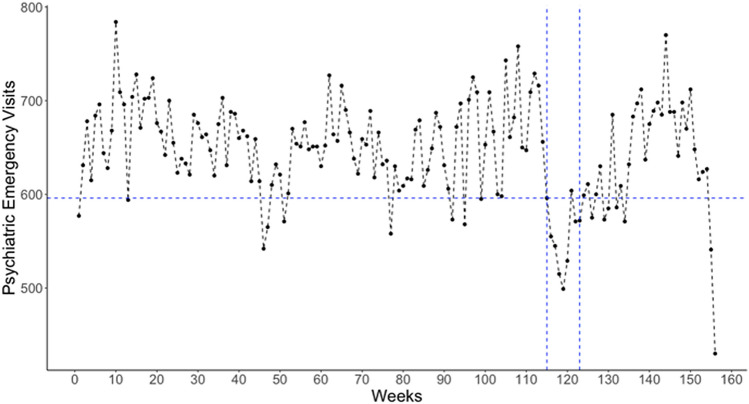
Count of Psychiatric Emergencies in LAC + USC Hospital over 156 weeks from Jan 5, 2018 to Dec 31, 2020. *Note* Initial set of societal restrictions (March 13 to May 8, 2020) shown with the blue dashed vertical lines. The left vertical line is at week 115 beginning on March 13, 2020 with 596 psychiatric emergency visits. The right vertical line is at week 123 beginning on May 8, 2020

Over the 156 weeks spanning Jan 5, 2018, to Dec 31, 2020, 98,888 psychiatric ED visits occurred at LAC + USC. Figure 1 plots the weekly count of these visits (mean = 646.24; standard deviation [SD] = 53.88). The basic plot shows a reduction in visits during the 1st set of societal restrictions (from week 115 to week 123) relative to the previous period. In addition, the lowest frequency of visits coincides with the last week of 2020, at the peak of the 2020–2021 winter surge of COVID-19 in LA.

**Fig. 2:**
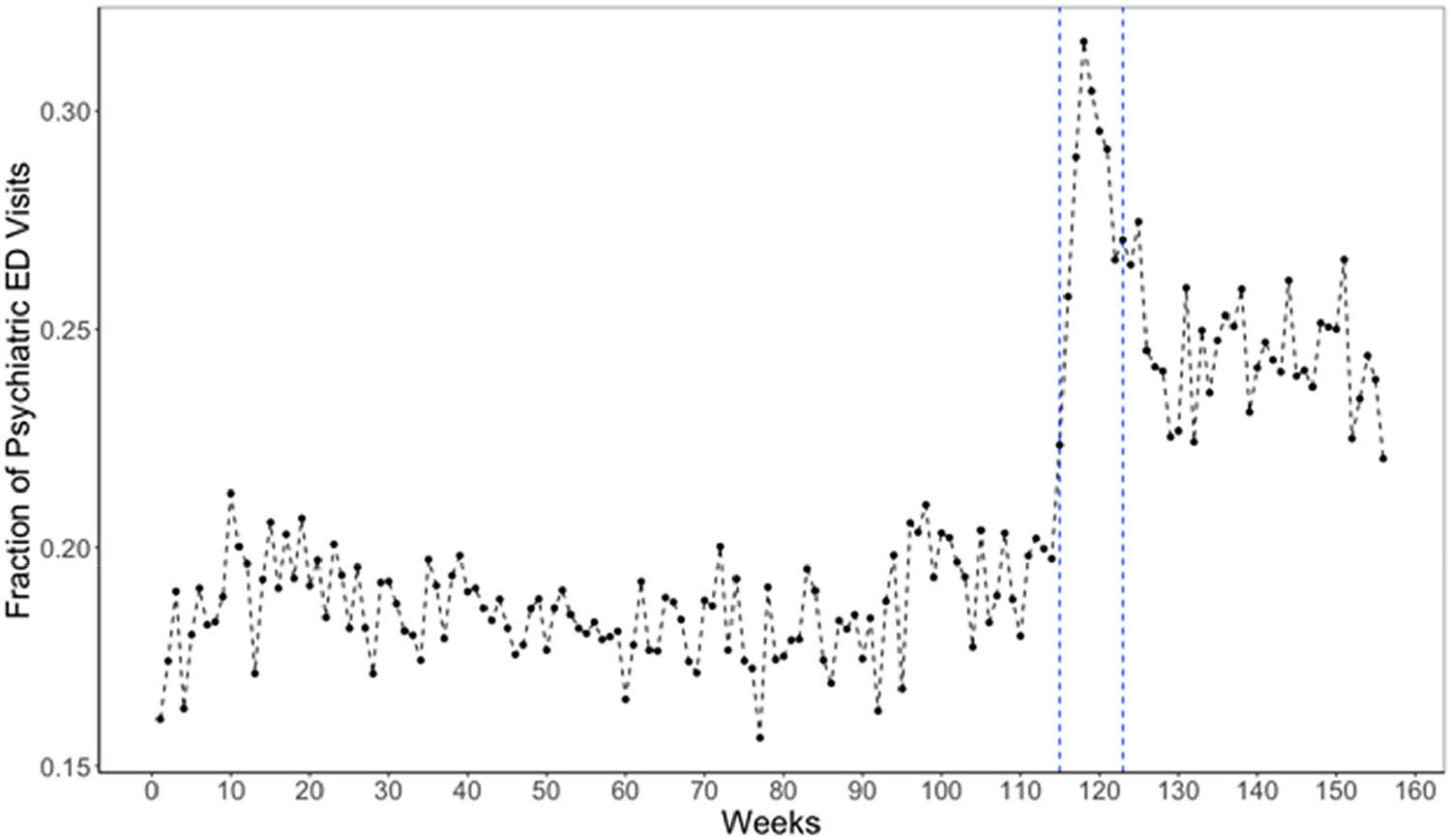
Proportion of ED visits classified as psychiatric in LAC + USC hospital over 156 weeks from Jan 5, 2018 to Dec 31, 2020. Initial set of societal restrictions (March 13 to May 8, 2020) shown with the blue dashed vertical lines

Figure 2 plots the weekly proportion of ED visits classified as psychiatric in LAC + USC over 156 weeks. The plot shows an increase in this proportion during the eight weeks of the 1st societal shutdown in LA County. After the relaxation of shutdown, the weekly proportion of ED visits classified as psychiatric declines but still exceeds pre-pandemic levels.

To examine the “shutdown” hypothesis, time-series routines indicate that high (or low) values of psychiatric ED visits were remembered, albeit in diminishing amounts, one and four weeks later. After the inclusion of autocorrelation error terms (i.e., AR1 and AR4 parameters), the weekly mean in psychiatric ED visits is zero. Interrupted time-series results (Table 1) indicate 78.95 fewer than expected psychiatric ED visits per week during the 1st set of societal restrictions (standard error [SE] = 24.69, p < 0.001). Summation of this reduction over the eight weeks of initial restrictions totals 711 fewer psychiatric ED visits than expected, which equates to a 12.03% reduction below mean levels.

**Table 1:** Time Series Results of Psychiatric Emergencies in LAC + USC Hospital over 156 weeks from Jan 5, 2018 to Dec 31, 2020 as a function of the 1^st^ COVID-19 Shutdown, the COVID-19 Rebound Period, and Autocorrelation

	Before COVID-19 restrictions		Including COVID-19 restrictions		COVID-19 restrictions and thereafter	
	Coefficient	SE	Coefficient	SE	Coefficient	SE
Constant	656.935	7.320***	648.666	8.434***	646.439	10.754***
1st Shutdown period			− 78.048	24.694***		
Rebound period					− 13.409	20.742
Time-series parameters						
AR1	0.276	0.092***	0.431	0.081***	0.523	0.076***
AR4	0.259	0.094***	0.238	0.085***	0.246	0.084***
Time period analyzed	Jan 5 2018 to March 12, 2020		Jan 5 2018 to Dec 31, 2020		Jan 5 2018 to Dec 31, 2020	

*p < 0.05, two-tailed; **p < 0.01, two-tailed; ***p < 0.001, two-tailed

We then inspected whether psychiatric ED visits “rebounded” after the first period of COVID-19 societal restrictions ended (Table 1). The coefficient for this time frame (i.e., May 9th to Dec 31, 2020) does not reach statistical detection above expected values (coef: − 13.409, SE = 20.742, p = 0.53). We then examined if any week post May 9th 2020, showed a positive “rebound” outlier in that its value exceeded the upper detection interval (set at 95% interval); we found none (Chen & Liu, 1993). The only outlier identified after May 9th, 2020, is negative and coincides with the peak of the 2020–2021 COVID-19 winter surge in LA (i.e., Dec 25th to Dec 31st, 2020).

**Fig. 3:**
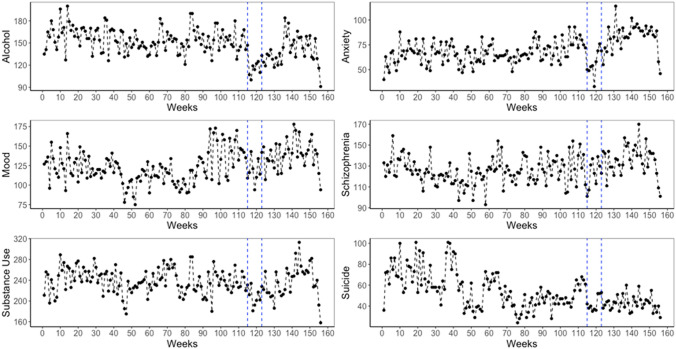
Counts of Psychiatric Emergencies by Subtype in LAC + USC Hospital over 156 weeks from Jan 5, 2018 to Dec 31, 2020

Given the discovered reduction in psychiatric ED visits during initial societal restrictions, we explored which type of visit, if any, accounted for a detectable share of this reduction. This exploration indicates that alcohol use disorder, followed by substance use disorder, and anxiety disorder, in diminishing magnitude of effect, each show reductions in ED visits during initial societal restrictions (Table 2 and Fig. 3 in Appendix 2). By contrast, ED visits for schizophrenia and suicidal ideation/attempt show no difference from expected levels. However, we caution the reader against over-interpreting cause-specific visits owing to the overlapping nature of these diagnoses with other psychiatric diagnoses on the ED record.

**Table 2:** Time series results of specific causes of psychiatric emergencies in LAC + USC hospital over 156 weeks from Jan 5, 2018 to Dec 31, 2020 as a function of the 1st COVID-19 shutdown and autocorrelation

	Substance-related disorders		Schizophrenia and other psychotic disorders		Mood disorders		Alcohol-related disorders		Anxiety disorders		Suicide and intentional self-inflicted injury	
	Coef	SE	Coef	SE	Coef	SE	Coef	SE	Coef	SE	Coef	SE
Constant	238.32	2.95***	127.45	1.54***	124.96	4.37***	150.54	2.54***	70.98	2.02***	52.76	6.13***
1st shutdown	− 22.56	11.43*	− 6.19	6.01	− 8.96	8.87	− 23.66	7.57**	− 11.50	5.95	− 5.89	7.73
Time-series parameters												
AR1	0.32	0.08***	0.29	0.08***	0.21	0.08*			0.37	0.08***	0.89	0.05***
AR3					0.22	0.07**						
AR4					0.37	0.07***			0.29	0.08**		
AR5							0.30	0.08***				
MA1							− 0.28	0.08***			0.43	0.09***

*p < 0.05, two-tailed; **p < 0.01, two-tailed; ***p < 0.001, two-tailed

## Discussion

The early stages of the COVID-19 pandemic in the US dramatically reduced help-seeking for health care. Prior work suggests that this reduction also occurred among psychiatric ED visits (Hartnett et al., 2020; Holland et al., 2021; Simpson et al., 2021). We examined this potential reduction during initial stay-at-home orders and, importantly, whether psychiatric ED visits showed a rebound above pre-pandemic levels following the lifting of stay-at-home orders. In one of the largest hospitals in the US, we find a significant but acute reduction in psychiatric ED visits during the first stay-at-home order. Alcohol use disorder, followed by substance use disorder, and to a lesser extent anxiety disorder account for the reduction of psychiatric ED visits after the shutdown. This reduction, however, was not followed by an unexpectedly high level of compensatory visits. Instead, psychiatric emergencies returned to but did not exceed pre-pandemic levels when stay-at-home orders ended. Findings appear consistent with the notion that the reduction of help-seeking for psychiatric care in the early stage of the pandemic did not exacerbate conditions to the point of creating a “psychiatric pandemic” of ED visits later in 2020.

Our analysis makes several key contributions. First, and perhaps most important, we formally test the possibility of a rebound in psychiatric ED visits post-shutdown. Second, unlike earlier work, we use rigorous time-series methods to control for strong confounding by autocorrelation. Third, we examine a well-delineated period of “stay-at-home” orders that lasted eight weeks and permit a straightforward test of help-seeking changes. Another strength of the study involves the exploration of different ED subtypes to identify which psychiatric conditions responded more sensitively to the various stages of the COVID-19 pandemic in 2020. Subtype results (Table 2 and Fig. 3 in Appendix 2) cohere somewhat with prior literature in that ED visits for alcohol use and substance use (and, to a lesser extent, anxiety) accounted for the large share of the “shelter in place” decline in psychiatric ED visits (Ferrando et al., 2021; McAndrew et al., 2021).

Limitations include that we did not have access to specific age groups at the time of our tests. We also lack ED data for 2021. Our study only includes nine months of pandemic data, which is a relatively short period in the ongoing post-pandemic and might not capture the pandemic’s impact on mental health in the long term. For example, youth may remain a high-risk group in 2021 due to continued school closures and online study formats (Clemens et al., 2020; Lee, 2020). In addition, we lacked information on other modalities of care (including telemedicine and primary care visits) that would complement emergency help-seeking. The literature finds that telemedicine accounted for 5% of mental health/substance use disorders’ outpatient visits pre-pandemic and 83.3–83.5% since the COVID-19 surge in Massachusetts, which indicates that telemedicine might serve as a key treatment option during the pandemic (Yang et al., 2020).

Results for the “shutdown” analysis appear consistent with literature in the US (Ferrando et al., 2021; Holland et al., 2021; Simpson et al., 2021) and elsewhere (Capuzzi et al., 2020; Gonçalves-Pinho et al., 2021; Hoyer et al., 2021; Rodriguez-Jimenez et al., 2021). Researchers found a dramatic decrease in psychiatric emergency visits during COVID-19 emergency state and lockdown in Italy (Capuzzi et al., 2020), Portugal (Gonçalves-Pinho et al., 2021), Spain (Rodriguez-Jimenez et al., 2021), and Germany (Hoyer et al., 2021). Reasons for the reduction in Europe may include reluctance to seek medical care during the high infectious rate period. Researchers also found reductions in visits due to alcohol and substance use disorder, anxiety and depression in the emergency setting (Ferrando et al., 2021; McAndrew et al., 2021). However, we note that LA experienced very low levels of COVID-19-related hospitalizations during the 1^st^ stage of restrictions.

After lifting stay-at-home orders, we did not observe a “rebound” of psychiatric emergencies that exceeded pre-pandemic levels. The result coheres with reports from insurance companies that overall health care utilization and spending did not rebound to the pre-pandemic levels after the second quarter of 2020 (Cox et al., 2021; Gallagher et al., 2021; Mehrotra et al., 2021; Rhyan et al., 2020). That prior work, however, does not focus on psychiatric ED visits. In the broader LA region, several adjacent hospitals report a rising share of ED visits classified as psychiatric, but none report an increase in the number of psychiatric ED visits during the 1st set of societal restrictions or during the potential “rebound” period (“Report Center—HCAI”, 2020). This circumstance indicates that our LAC + USC results likely reflect the county's broader system of ED care. This pattern of results, which to our knowledge has not appeared previously in the literature, indicates that an increase in psychiatric care teams to meet higher ED demand may not be warranted in the long run.

We speculate that the lack of a “rebound” effect in psychiatric ED visits following COVID-19 related “shelter in place” restrictions indicates that foregoing psychiatric care provided in the ED does not adversely affect at least a subset of patients. In addition, our pattern of results suggests that the true incidence of a subset of psychiatric symptoms may have declined during COVID-19. Work outside the US, for instance, reports a “honeymoon” period in which, in the short term, certain conditions (e.g., anxiety, suicidal ideation/attempt, alcohol use disorder, mood disorder) may have abated in light of larger shared concerns around the impending pandemic (Andersen et al., 2021; Brülhart et al., 2021). This explanation, however, does not preclude the possibility that specific subgroups (e.g., youth, senior citizens living alone) disproportionately showed worse mental health symptoms during COVID-19. All of these claims require careful formulation and follow-up research before being taken as anything other than informed speculation.

Whereas the absolute number of psychiatric ED visits decreased during the 1st societal shutdown, the weekly proportion of ED visits classified as psychiatric rose substantially and stayed elevated after the relaxation of shutdown (Fig. 2). Findings indicate that the demand for psychiatric ED services fell less steeply during COVID-19 than did the demand for *non*-psychiatric ED services. This result coheres with previous studies (Holland et al., 2021). Future work may identify the particular subtypes of non-psychiatric care that were averted during the COVID-19 shelter-in-place period and thereafter.

We note two important caveats to our population-level study. First, specific age groups (e.g., youth) may have experienced a surge in emergency visits, especially in light of the extended suspension of in-person schooling in LA County. Second, studies elsewhere (in Kentucky and Ohio) find increased ED visits for suspected opioid overdose after the declaration of a COVID-19 national emergency (Root et al., 2021). We therefore encourage replication efforts in other regions, as well as further identification of at-risk subgroups with unmet psychiatric needs.

## Conclusion

Future research may focus on examining age differences of psychiatric emergency visits during the pandemic, especially for adolescents. Moreover, we encourage additional analyses to investigate changes through 2021 and incorporate other data, such as help hotline calls and primary care visits, to provide context. Furthermore, investigation of the extent to which complementary services (e.g., telemedicine) averted the need for ED help-seeking, during the societal restrictions, appears warranted.

## Appendix 1

See Table 3.

**Table 3:** CCS codes for psychiatric ED visit diagnosis ranging from 650 to 670

CCS code	Diagnosis
650	Adjustment disorders
651	Anxiety disorders
652	Attention-deficit, conduct, and disruptive behavior disorders
653	Delirium, dementia, and amnestic and other cognitive disorders
654	Developmental disorders
655	Disorders usually diagnosed in infancy, childhood, or adolescence
656	Impulse
657	Mood disorders
658	Personality disorders
659	Schizophrenia and other psychotic disorders
660	Alcohol-related disorders
661	Substance-related disorders
662	Suicide and intentional self-inflicted injury
663	Screening and history of mental health and substance abuse codes
670	Miscellaneous mental health disorders

Consistent with the federal government’s classification scheme, we identified a psychiatric ED visit using Clinical Classification Software (CCS). CCS categorization combines individual *ICD-9* diagnoses into clinically meaningful categories, among which CCS codes 650 to 670 receive a mental health-related diagnosis. We adopt these definitions, used by the Healthcare Cost and Utilization Project (HCUP) and other federal databases, in our analysis. We considered the ICD-9 code as a psychiatric ED visit if *any* diagnosis for a visit listed a corresponding CCS code ranging from 650 to 670. These CCS codes include but are not limited to the following conditions: mood, anxiety, schizophrenia, and behavioral disorders; suicide attempts; self-harm; and alcohol and substance-related disorders. We include alcohol and substance use disorders because the federal government classifies them under psychiatric conditions in the CCS

## Appendix 2

See Fig. 3.

## Declarations

### Standards of Reporting

None of the guidelines is applicable to this study.
